# Supplementary material for: Acute exogenous acyl-GIP treatment enhances lipid handling and fatty acid oxidation by involving brown fat
Source: EMBO Rep. 2025 Sep 22;26(21):5154–71. doi: 10.1038/s44319-025-00582-7 (PMC12592529; doi:10.1038/s44319-025-00582-7)
Supplement: Supplementary file 8 — Expanded View Figures [file 44319_2025_582_MOESM8_ESM.pdf]

Expanded View Figures

Figure EV1. Assessment of GIPR agonism and GIPR antagonism on glucose and lipid tolerance in male mice.

Comparison in (**A**) body weight, (**B**) body composition and (**C**) 4-h fasting blood glucose levels between lean (regular chow diet; RCD) and obese (60% high-fat diet; HFD) 20-week-old male C57BL/6 mice ($n = 15$–16 for RCD; $n = 32$–36 for HFD). Determination of the efficacy of acyl-GIP during a lipid tolerance test (LTT; (**D**) and intraperitoneal glucose tolerance test (IPGTT; (**E**) Acute acyl-GIP (1 nmol/kg) administration lowers glucose excursions during an intraperitoneal glucose tolerance test (IPGTT) in (**F**) lean mice (20-weeks old, $n = 8$ for PBS and acyl-GIP) and (**G**) obese mice (20-weeks old, $n = 8$ for PBS and acyl-GIP). The impact on glucose tolerance when both acyl-GIP (1 nmol/kg) and a GIPR antagonist (1500 nmol/kg) is administered in (**H**) 20–30-week-old obese male mice ($n = 3$–7 per treatment group). The effects of acyl-GIP on 20-week-old lean (**I**) whole-body *Gipr* wild-type (*Gipr*$^{+/+}$; PBS and acyl-GIP, $n = 4$) and (**J**) whole-body *Gipr knockout* mice (*Gipr*$^{-/-}$; PBS and acyl-GIP, $n = 5$). (**K**) A comparison of various adipose tissue depot *Gipr* mRNA expression between RCD and HFD-fed mice. All values made relative to RCD BAT *Gipr* levels ($n = 6$–7 for RCD and HFD mice). Experiments were repeated minimum 2 times with each animal receiving either PBS or GIP in a cross-over design study and data was pooled with 2 cohorts of animals. Except for data described in K, as the experiments were terminal. Two-way Repeated Measures ANOVAs or linear mixed effects analysis were performed assessing effects of treatment and time, with a Šídák's multiple comparisons test to compare between treatments at a given timepoint. One-way ANOVAs with a Dunnett's post hoc test were used to assess AUCs (area under the curve) compared to PBS group. Student unpaired *T* tests were used to compare PBS and GIP treatment groups, and to compare RCD and HFD-fed mice. *$P < 0.5$; **$P < 0.01$; ***$P < 0.001$; ****$P < 0.0001$. $ Significant difference between 1 and 10 nmol/kg groups and PBS ($P < 0.05$). # Significant differences between 10 nmol/kg and PBS ($P < 0.05$). Data are presented as mean ± SEM. BAT, brown adipose tissue. iWAT, inguinal white adipose tissue. eWAT, epididymal white adipose tissue. rWAT, retroperitoneal white adipose tissue. For exact *P* values, please refer to Dataset EV1. Source data are available online for this figure.

 © The Author(s)

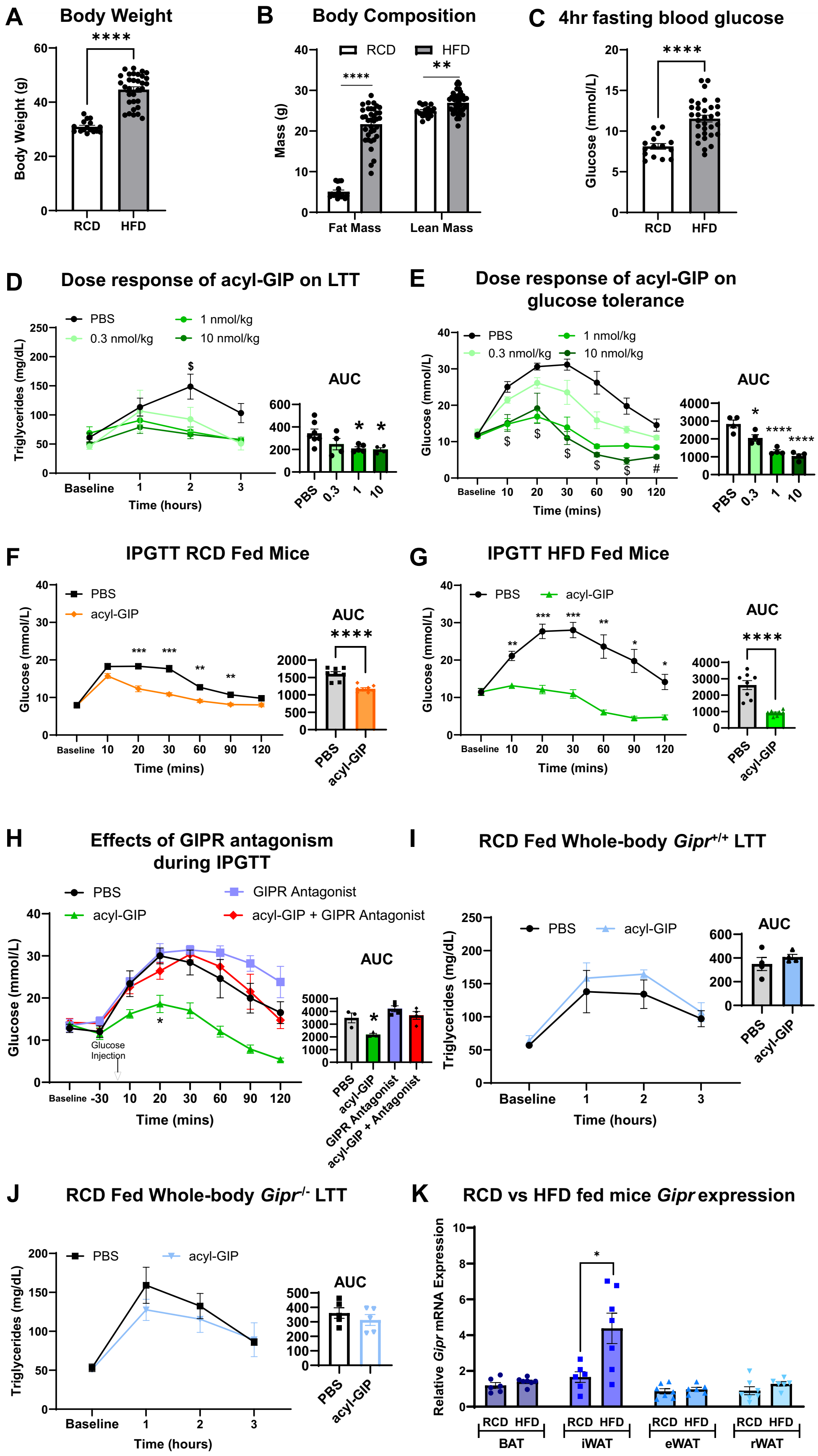
A Body Weight
B Body Composition
C 4hr fasting blood glucose
D Dose response of acyl-GIP on LTT
E Dose response of acyl-GIP on glucose tolerance
F IPGTT RCD Fed Mice
G IPGTT HFD Fed Mice
H Effects of GIPR antagonism during IPGTT
I RCD Fed Whole-body Gipr+/+ LTT
J RCD Fed Whole-body Gipr-/- LTT
K RCD vs HFD fed mice Gipr expression

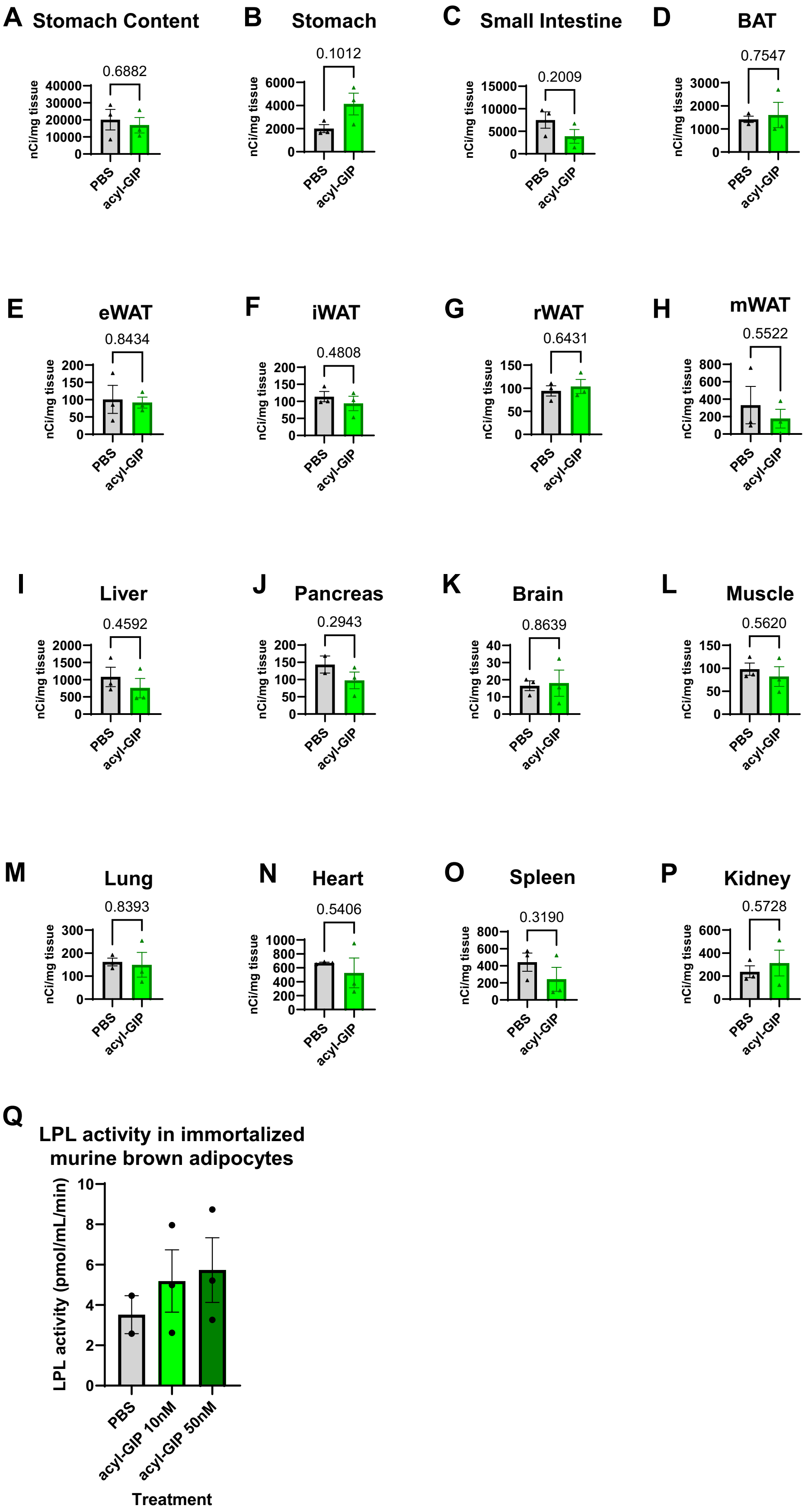
A Stomach Content
0.6882
B Stomach
0.1012
C Small Intestine
0.2009
D BAT
0.7547
E eWAT
0.8434
F iWAT
0.4808
G rWAT
0.6431
H mWAT
0.5522
I Liver
0.4592
J Pancreas
0.2943
K Brain
0.8639
L Muscle
0.5620
M Lung
0.8393
N Heart
0.5406
O Spleen
0.3190
P Kidney
0.5728
nCi/mg tissue
PBS
acyl-GIP
Q LPL activity in immortalized murine brown adipocytes
LPL activity (pmol/mL/min)
PBS
acyl-GIP 10nM
acyl-GIP 50nM
Treatment

© The Author(s)

Figure EV2. Values for ^{3}H-palmitic acid uptake 2 h following an oral lipid challenge in male obese mice fed a 60% HFD for 12 weeks, and lipoprotein lipase (LPL) activity of day 8 differentiated murine brown adipocytes.

Lipid phase ^{3}H-activity relative to tissue mass in a variety of mouse tissues used to calculate relative % distribution for Fig. 2G. $n = 3$ for PBS and acyl-GIP. Experiments were completed in 1 cohort of mice as these experiments were terminal (**A–P**). Student unpaired *T* tests were used to compare PBS and GIP treatment groups. (**Q**) 8 day differentiated immortalized murine brown adipose tissue cells exposed to various concentrations of acyl-GIP to assess lipoprotein lipase (LPL) activity. Each data point represents 1 biological replicate ($n = 2$ for pbs, $n = 3$ for 10 and 50 nM acyl-GIP). One-way ANOVA with a Dunnett's post hoc test was used to compare PBS to all GIP treatments. Data are presented as mean ± SEM. Source data are available online for this figure.

© The Author(s)

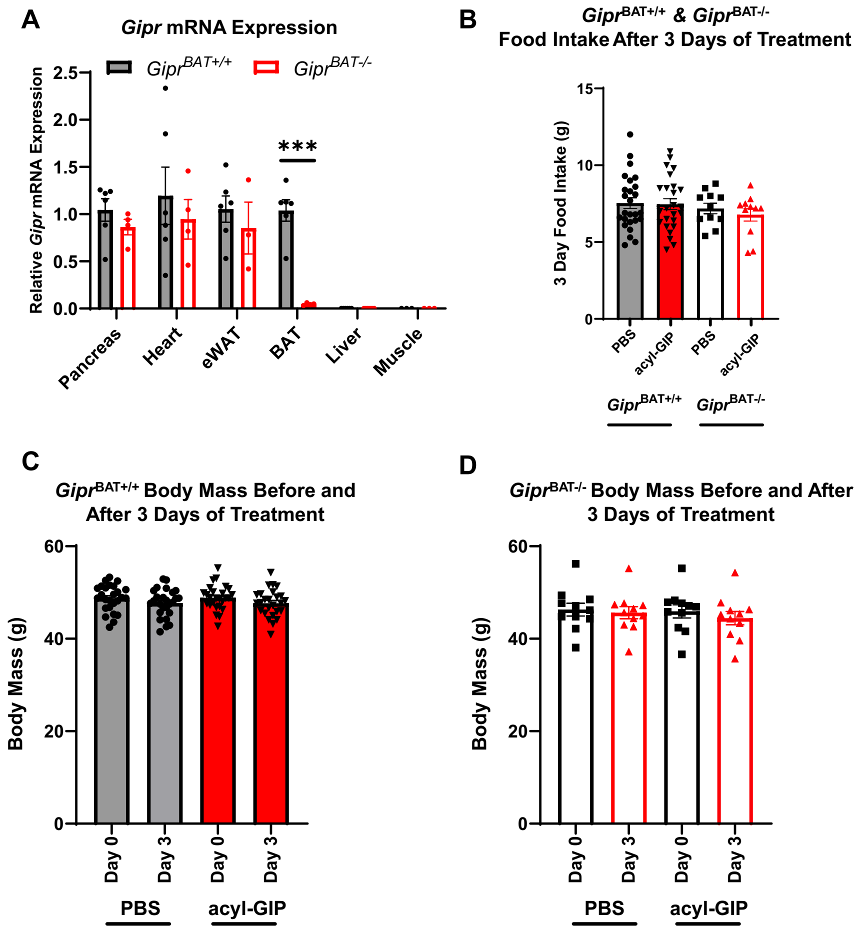

Figure EV3. BAT specific GIPR knockout mouse model.

(**A**) *Gipr* gene expression in a variety of tissues, confirming tissue specific knockout in the BAT ($Gipr^{BAT+/+}$; $n = 3$–6, $Gipr^{BAT-/-}$; $n = 3$–4). (**B**) After 3 consecutive days of PBS of acyl-GIP (1 nmol/kg) treatment, there was no change in the cumulative food intake of obese male $Gipr^{BAT+/+}$ ($n = 26$) and $Gipr^{BAT-/-}$ ($n = 11$) mice. After 3 consecutive days of PBS of acyl-GIP (1 nmol/kg) treatment, no change in body mass was observed in (**C**) $Gipr^{BAT+/+}$ or (**D**) $Gipr^{BAT-/-}$ obese mice ($Gipr^{BAT+/+}$; $n = 26$, $Gipr^{BAT-/-}$; $n = 11$). Experiments were repeated minimum 2 times with each animal receiving either PBS or GIP in a cross-over design study and data was pooled with 3 cohorts of animals. $^{***}P < 0.001$. Student unpaired *T* tests were used to compare genotypes, treatment groups, and time on treatment. Data presented as mean ± SEM. For exact *P* values, please refer to Dataset EV1. Source data are available online for this figure.

 © The Author(s)
